# Inclusion of Grape Pomace in Finishing Cattle Diets: Carcass Traits, Meat Quality and Fatty Acid Composition

**DOI:** 10.3390/ani12192597

**Published:** 2022-09-28

**Authors:** Frances A. Arend, Gordon K. Murdoch, Matt E. Doumit, Gwinyai E. Chibisa

**Affiliations:** Department of Animal, Veterinary and Food Sciences, University of Idaho, Moscow, ID 83844, USA

**Keywords:** finishing cattle, grape pomace, meat fatty acid composition, product quality

## Abstract

**Simple Summary:**

Grape pomace, a by-product of winemaking, could be used as feed for beef cattle as it provides essential nutrients like fiber and protein. Grape pomace also contains bioactive compounds like condensed tannins, which have been shown to improve meat quality primarily in lambs. Since this information was still lacking, the present study evaluated the effects of feeding a high amount of grape pomace to finishing cattle on quality attributes (e.g., shelf life) and the fatty acid composition of beef. Feeding a high amount of grape pomace to finishing cattle reduced lipid oxidation and increased the meat content of several fatty acids linked to positive health outcomes in humans (e.g., total polyunsaturated fatty acids). These findings suggest that dietary inclusion of grape pomace in finishing cattle diets could enhance the sensory quality of beef and the health value of beef lipids.

**Abstract:**

Because of its high content of polyphenolic compounds, dietary inclusion of grape pomace (GP) in finishing cattle diet could possibly enhance product quality and the health value of beef lipids. Therefore, the aim of this study was to evaluate the effects of feeding a high amount of grape pomace in finishing cattle diets on carcass traits, product quality, and fatty acid (FA) composition of beef. Jersey × Holstein crosses (n = 24) were fed either a typical finishing diet (CON) or a finishing diet containing 58% grape pomace (DM basis; HGP). Following the feeding period, animals were harvested, and carcass traits measured. *Longissimus lumborum* (LL) and *semimembranosus* (SM) muscle were then collected from each carcass for sensory quality evaluation and FA profile analysis. Hot carcass weight, backfat thickness, and preliminary and final yield grades were greater (*p* ≤ 0.04) for CON than HGP steers. However, there was no diet effect on rib eye area (REA), kidney, pelvic, and heart (KPH) fat, and marbling. Feeding the HGP compared to CON diet reduced lipid oxidation of LL and SM steaks over time; the malondialdehyde (MDA) concentration, which did not differ on d 0 and 2 of 8-d simulated retail display, was lower on d 4, 6 and 8 for HGP than CON steers (treatment × day of simulated display interaction; *p* < 0.01). Brightness (L* values) and redness (b*) were greater for LL steaks from HGP than CON steers on most days of simulated display (treatment × day of simulated display interaction; *p* < 0.01). In addition, the LL and SM muscle content of several FA linked to positive health outcomes in humans including 18:2 *n*-6, 18:2 *c*9*t*11, total conjugated linoleic acid (CLA) and total polyunsaturated fatty acid (PUFA) was also greater (*p* ≤ 0.02) for steers fed the HGP compared to the CON diet. In summary, current findings suggest that although it could possibly limit growth performance, feeding a high amount of grape pomace to finishing cattle could enhance both the sensory quality and the health value of beef lipids, which are key in increasing consumer acceptability of beef.

## 1. Introduction

The use of agro-industry by-products as cattle feed could be effective in reducing both the competition between food and feed, and the environmental cost of beef production [1]. In the Pacific Northwest region, grape pomace (GP) is one such by-product that has appeal as cattle feed due to its ease of availability and lower cost relative to traditional feed ingredients including corn grain. Besides providing essential nutrients including fiber and fat, grape pomace also contains polyphenolic compounds such as proanthocyanidins (condensed tannins; CT) that include catechin, epicatechin, and procyanidins, which could potentially enhance beef quality. However, to the best of our knowledge, the impact of feeding grape pomace to finishing cattle on product quality and composition remains to be fully evaluated.

Meat attributes including marbling, color, and appearance are key determinants of consumer acceptability of beef. However, the high degree of marbling that is currently desired as it enhances the eating experience, also increases the probability of the occurrence of lipoperoxidation, which compromises sensory properties [2]. Therefore, limiting or delaying lipid oxidation is vital as it enhances quality by reducing sensory spoilage, including meat discoloration and the development of rancid flavors and odors [3]. Because of their antioxidant properties, increased intestinal absorption and transfer of polyphenolic compounds into meat when GP is fed to finishing cattle could potentially enhance oxidative stability of beef products [4]. In studies with finishing lambs [5,6], feeding up to 2.5% (DM basis) grape seed extract either reduced or prevented the accumulation of volatile lipo-oxidation compounds such as heptanal, 2-nonenal, 2-propanone and dimethylsulfide in *longissimus* muscle, suggestive of increased lipid stability. Moreover, dietary inclusion of 2.5% (DM basis) grape seed extract in finishing lamb diets reduced lipid oxidation [−15.5%; mg malondialdehyde (MDA)/kg] in *longissimus* muscle over 7 d of simulated retail display [7]. However, the effects of feeding grape pomace to finishing cattle on the sensory properties including lipid oxidation and color stability of beef remain to be fully evaluated. 

In addition to sensory quality, healthiness of beef is increasingly an important attribute that shapes consumer acceptability [8]. Although beef is a good source of various nutrients including iron and zinc, there are concerns about the saturated fatty acid (SFA), including palmitic (16:0) acid it contains, whose consumption in high amounts has been linked to increased risk for chronic diseases, including cancer and cardiovascular disease (CVD; [9]). Therefore, there is continued interest in the use of dietary strategies to reduce the concentration of the undesirable SFA and/or increase the accumulation of the desirable polyunsaturated fatty acids (PUFA) including linoleic (18:2 *n*-6) and α-linolenic (18:3 *n*-3) acids in meat. When fed to ruminants, polyphenolic compounds such as CT can reduce microbial biohydrogenation of dietary PUFA [10,11]. This in turn increases the ruminal accumulation and subsequent transfer into meat of the desirable dietary PUFA (e.g., 18:2 *n*-6, 18:3 *n*-3) and biohydrogenation intermediates (e.g., 18:2 *c*9*t*11) at the expense of the undesirable fatty acids (e.g., 18:0 and 18:2 *t*10*c*12). Feeding grape pomace to dairy ewes and cows increased the milk content of 18:2 *n*-6, 18:1 *t*-11, total PUFA, and 18:2 *c*9*t*11, and also reduced the content of the undesirable SFA [12,13,14]. However, there is limited information on the impact of feeding grape pomace in finishing cattle on the FA profile of beef. Therefore, the objective of the current study was to evaluate the effect of feeding a high amount of grape pomace to finishing cattle on meat quality (e.g., color, and shelf life) and the FA composition of beef. We hypothesized that dietary inclusion of a high amount of grape pomace during the finishing period increases the sensory quality of beef due to the transfer of polyphenolic compounds into meat, and enhances the health value of beef lipids due to the increased accumulation of desirable FA at the expense of the undesirable FA in meat.

## 2. Material and Methods

All animal care and handling procedures followed protocols preapproved by the University of Idaho Animal Care and Use Committee (protocol #: 2016-37) prior to initiation of study. 

### 2.1. Animals and Diets

A group of 24 crossbred (Jersey × Holstein) steers (mean ± SD, 301 ± 5.45 kg incoming BW) were raised on a commercial feedlot (Sunnyside, WA) for the entire finishing phase. At trial initiation, steers were vaccinated against Infectious Bovine Rhinotracheitis (IBR), Bovine Viral Diarrhea (BVD) Type 1 and Type 2, Parainfluenza3 (PI_3_), Bovine Respiratory Syncytial Virus (BRSV), 5 strains of *Leptospirosis*, including *hardjo-bovis*, and *Campylobacter* fetus (vibriosis) (Vista 5L5 SQ; Merck, Rahway, NJ, USA). Steers were also treated for parasites using ivermectin (Ivomec, Merial, Duluth, GA, USA), and implanted with 80 mg of trenbolone acetate and 16 mg estradiol (Revalor IS; Merck Animal Health, Whitehouse Station, NJ, USA). After approximately 91 d on feed, steers were reimplanted with 200 mg of trenbolone acetate and 40 mg estradiol (Revalor-XS, Merck Animal Health, Summit, NJ, USA) and treated with Titanium IBR-LP (Elanco Animal Health, Greenfield, IN, USA) for revaccination against bovine rhinotracheitis and the prevention of *Leptospira pomona*. On d 1, steers were randomly assigned to either a by-product-based finishing diet typically fed in the Pacific Northwest (CON; n = 12) or a finishing diet containing 58% (DM basis) ensiled grape pomace (HGP; n = 12) (Table 1) in a completely randomized design. The ensiled grape pomace partially replaced some of the energy sources including high moisture corn in the CON diet. Animals were fed for *ad libitum* intake and feed was offered twice a day. The target liveweight for slaughter was 530 kg and this led to CON and HGP steers being fed the finishing diets for 261 and 288 d, respectively. Average daily gain was 1.17 and 0.88 kg/d for CON and HGP steers, respectively.

Weekly feed samples were collected and dried at 55 °C for 72 h, and then sequentially ground through a 4- and 2-mm screen (Retsch Cutting Mill SM 200, Retsch, Haan, Germany). Ground samples were sent to Cumberland Valley Analytical Services (Hagerstown, MD, USA) for analysis. Samples were analyzed for DM and OM (method 942.05; [15]). Nitrogen (method 990.03; [15]) was determined by rapid combustion using a Macro Elemental nitrogen analyzer, and CP was calculated as nitrogen value was multiplied by 6.25. Acid detergent fiber (ADF) was determined per [16] method 973.18 and neutral detergent fiber (NDF) was determined using α-amylase and sodium sulfite. Ash was determined per [15] method 942.05. Crude fat was quantified per [16] method 2003.05 whereas fatty acids were measured as described by [17]. Feed samples were also analyzed for phenols (i.e., total phenols, tannin, and nontannin phenols) as described by [18]. Briefly, following ball grinding, free phenolics were extracted using ethanol, whereas acid extraction was used for total phenolics (free plus bound forms). Resultant extracts were then analyzed for phenolics using the Folin–Ciocalteu method. In addition, polyvinyl-polypyrrolidone was used to precipitate tannins in aliquots of the ethanolic or acidic extracts prior to analysis of nontannins using the Folin–Ciocalteu method. Tannins were then determined by difference. 

### 2.2. Carcass Data Collection

At the end of the feeding period, steers were weighed prior to transportation (3.5 h via semitrailer) to the harvest facility (University of Idaho Meat Science Laboratory). Upon arrival, steers were held approximately 2 to 6 h before harvest to allow for inspection by USDA Food Safety and Inspection Service (FSIS) personnel. Following harvest, carcasses were chilled for at least 24 h, and dressing percent, 12th rib fat thickness, preliminary yield grade, rib eye area (REA), kidney, pelvic and heart fat (KPH), final yield grade (YG), and marbling score were determined by trained University of Idaho personnel. 

### 2.3. Product Preparation 

At the end of processing, strip loins (Institutional Meat Purchase Specifications (IMPS) 180; [19]) and top (inside) round (IMPS 168; [19]) were collected from the left side of each carcass. The *longissimus lumborum* (LL) and *semimembranosus* (SM) were removed from the respective wholesale cuts, vacuum shrink packaged (7 × 12 in. Durashrink bags, Winpak Films, Senoia, GA, USA), and subsequently wet aged for 14 d at 0 °C. Following aging, 2.54 cm-thick steaks were cut and randomly assigned for evaluation of color (Hunter Mini-Scan) and lipid oxidation (Thiobarbituric acid reactive substances; T-BARS) over 8-d of simulated retail display, tenderness (Warner-Bratzler shear force; WBSF), and fatty acid (FA) composition.

### 2.4. Retail Color

Steaks used for retail color evaluation were weighed, placed in white Styrofoam trays with the freshly cut surface exposed, and overwrapped with an oxygen permeable PVC film (Koch Industries, Inc. #7500-3815; Wichita, KS, USA). Following blooming for at least 60 min, two objective color measurements were taken per steak using a Hunter MiniScan EZ (Restin, VA, USA); this represented d 0 of simulated retail display. Steaks were then displayed in a glass-fronted retail display case (Model GDM-69, True Manufacturing Co., O’Fallon, MO, USA) kept at 2 °C for 8 days. The display case used natural white Hg 40 W lights and the average light intensity was 409 lx. Daily hunter color measurements were then taken, and steaks were rotated after each measurement to minimize location effects. The Hunter MiniScan, which is equipped with a 25-mm diameter measuring window and a 10° standard observer was set to illuminant A, and Commission International de l’Eclairage (CIE) L* (lightness), a* (redness) and b* (yellowness) duplicate values were recorded. Calibration of the colorimeter was performed each day by measuring against black and white calibration tiles according to the manufacturer’s instructions. Subjective color measurements were also taken daily by 3 trained evaluators following Section 7 Appendix C of the American Meat Science Association guidelines [20]. Steaks were scored for discoloration (1 = none; 5 = extreme), amount of browning (1 = no evidence of browning; 6 = dark brown), and color uniformity (1 = uniform; 5 = extreme two-toning). Following the 8-d simulated retail display, steaks were weighed to determine retail fluid loss. 

### 2.5. Lipid Oxidation

Thiobarbituric acid reactive substances (TBARs) were analyzed on d 0, 2, 4, 6 and 8 of simulated retail display using the protocol provided in Section XI, Appendix O of the Meat Color Measurement Guidelines [20]. The end (~1 cm) of the steak was discarded before samples (~0.5 cm wide × 2.0 cm long × 1.27 cm thick) were taken from the top half of the steak avoiding the edge.

### 2.6. Cooking

Following retail display, weighed steaks were cooked on open-hearth broilers to an internal temperature of 40 °C before being turned and cooked to a final internal temperature of 71 °C. Hypodermic temperature probes (Omega Engineering Co., Stamford, CT, USA) coupled with a 12-channel scanning thermocouple thermometer (Digi-Sense, Cole-Parmer Instrument Co., Vernon Hills, IL, USA) were used to monitor the temperature. Following cooking, steaks were weighed again to determine percent cook loss, and then refrigerated overnight at 3 °C for subsequent WBSF analysis.

### 2.7. Warner–Bratzler Shear Force

Following cooking and refrigerated storage, six cores (1.27-cm diameter) were mechanically removed parallel with the muscle fiber orientation using a drill press-mounted coring device (GR Manufacturing, Manhattan, KS, USA) from each steak. Shear force was then determined by shearing each core (200 mm/min) perpendicular to the muscle fibers using a Warner–Bratzler shear machine (GR Manufacturing, Manhattan, KS, USA). The six shear values were averaged to determine a shear force (kg) value for each steak. 

### 2.8. Fatty Acid Analysis

Muscle (LL and SM) samples (5 g) for fatty acid analysis were freeze-dried and ground under dry ice before lipid extraction [21]. Following their formation from extracted lipids [22], methyl esters were analyzed using a gas-liquid chromatograph (Hewlett-Packard 6890 series with auto injector; Agilent Technologies, Inc., Palo Alto, CA, USA). A CP-Sil 88 fused-silica capillary column (100 m × 0.25 mm, Chrompack, Raritan, NJ, USA) and programmed temperature gradient were used for peak seperation. Gas pressure of H_2_ (carrier gas) was fixed, with injector and detector temperatures set to 255 °C. The oven temperature gradient was as follows: (1) initial increase from 70 °C to 175 °C (rate of 4 °C/min) and kept constant for 3 min after sample injection, (2) increase to 185 °C (rate of 1 °C/min) and kept constant for 20 min, (3) increase to 215 °C (rate of 3 °C/min) and then 240 °C (rate of 10 °C/min) and kept constant for 5 min, and (4) return to 70 °C. Pure standards (Matreya, Inc., Pleasant Gap, PA, USA) were used to identify peaks and weight percent calculations were based on a reference butter oil sample (CRM 164, European Community Bureau of Reference, Brussels, Belgium).

### 2.9. Statistical Analysis

Data were analyzed as a completely randomized design using the MIXED procedure of SAS (SAS Inst. Inc., Cary, NC, USA). Animal was the experimental unit, and diet and day of simulated retail display, and their interaction were fixed variables. All color and TBARS measurements were analyzed accounting for repeated measures. Residual distributions were evaluated for normality and homoscedasticity and data sets not meeting the model assumptions (residual normality and homogeneity of variance) were transformed (log or square root) as needed prior to analysis. Differences in least squares means were compared using the DIFF option. Significance was declared at *p* < 0.05 with tendencies discussed at 0.05 ≤ *p* ≤ 0.10. 

## 3. Results

### 3.1. Dietary Chemical Composition

Diets were iso-nitrogenous (average of 15.8% CP; Table 1) by design. However, both NDF and ADF content were over 1-fold greater for the HGP than CON diet. On the other hand, dietary net energy of gain was over 2-fold greater for the CON compared to the HGP diet. Dietary stearic and oleic acid concentrations were greater for the CON than HGP diet, whereas the linoleic acid concentration was greater for the HGP than CON diet (Table 2).

### 3.2. Carcass and Meat Quality

Although hot carcass weight was greater (*p* < 0.01) for the CON than HGP steers, there was no diet effect (*p* = 0.30) on dressing percentage (Table 3). Backfat thickness, and preliminary and final yield grades were greater (*p* ≤ 0.04) for the CON than HGP steers. However, there was no diet effect (*p* ≥ 0.14) on REA, KPH, and marbling. Fluid loss for LL steaks tended (*p* = 0.057) to be greater for steers fed the CON compared to the HGP diet (Table 4). However, cook loss and WBSF for LL steaks did not differ (*p* ≥ 0.35) across diets. Fluid and cook loss for SM steaks were greater (*p* < 0.01) for steers fed the HGP than CON diets. However, WBSF for SM steaks did not differ (*p* = 0.30) across diets.

### 3.3. Lipid Oxidation

**Figure 1:**
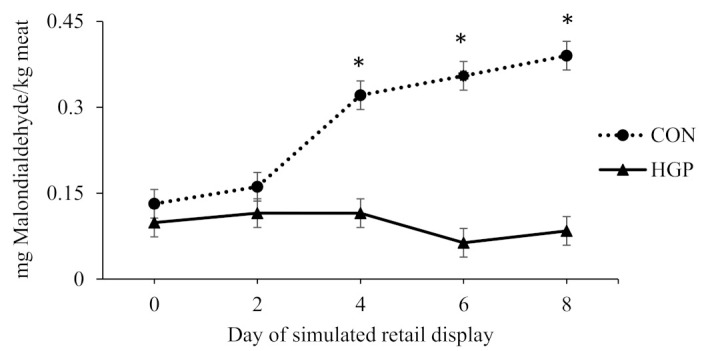
Lipid oxidation over 8 d of simulated retail display for striploin (*longissimus lumborum*) steaks from cattle fed a typical co-product-based finishing diet (CON) compared to a diet containing 58% (DM basis) grape pomace (HGP). Pooled SEM = 0.0257. Diet, *p* < 0.01; day of simulated retail display, *p* < 0.01; diet × day of simulated retail display, *p* < 0.01. Error bars represent the standard deviation. * Significant difference between CON and HGP at *p* < 0.05.

**Figure 2:**
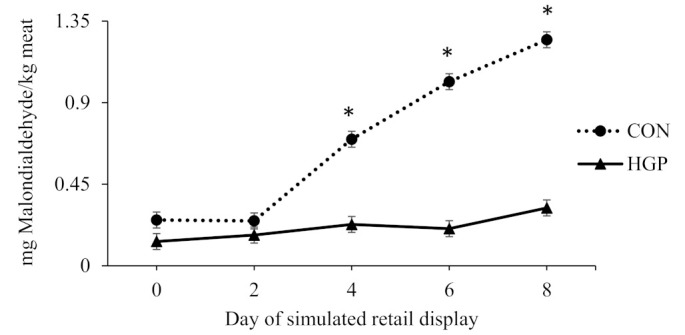
Lipid oxidation over 8 d of simulated retail display for top round (*semimembranosus*) steaks from cattle fed a typical co-product-based finishing diet (CON) compared to a diet containing 58% (DM basis) grape pomace (HGP). Pooled SEM = 0.0257. Diet, *p* < 0.01; day of simulated retail display, *p* < 0.01; diet × day of simulated retail display, *p* < 0.01. Error bars represent the standard deviation. * Significant difference between CON and HGP at *p* < 0.05.

There was a diet × day of simulated retail display interaction (*p* < 0.01) for TBARS values for LL steaks; although it did not differ on d 0 and 2 of simulated retail display, the MDA concentration was greater on d 4, 6 and 8 for CON than HGP steers (Figure 1). There was also a diet × day of simulated retail display interaction (*p* < 0.01) for TBARS values for SM steaks; although it did not differ on d 0 and 2 of simulated retail display, the MDA concentration was greater on d 4, 6 and 8 for CON than HGP steers (Figure 2). 

### 3.4. Color Stability

**Figure 3:**
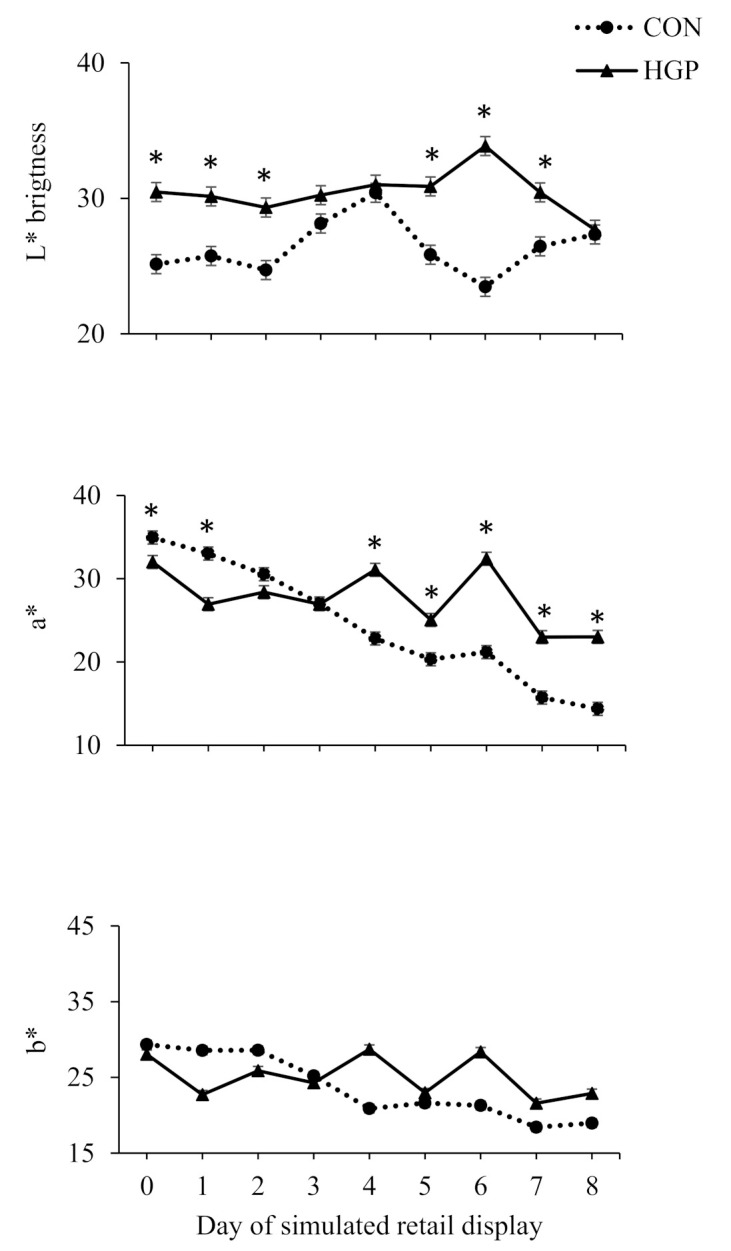
Hunter L*, a*, and b* color values over 8 d of simulated retail display for *longissimus* steaks from cattle fed a typical co-product-based finishing diet (CON) compared to a diet containing 58% (DM basis) grape pomace (HGP). L* color values pooled SEM = 0.7098. Diet, *p* < 0.01; day of simulated retail display, *p* < 0.01; diet × day of simulated retail display, *p* < 0.01. a* color values pooled SEM = 0.7797. Diet, *p* = 0.01; day of simulated retail display, *p* < 0.01; diet × day of simulated retail display, *p* < 0.01. b* color values pooled SEM = 0.5710. Diet, *p* = 0.08; day of simulated retail display, *p* < 0.01; diet × day of simulated retail display, *p* < 0.01. Error bars represent the standard deviation. * Significant difference between CON and HGP at *p* < 0.05.

There was a diet × day of simulated retail display interaction (*p* < 0.01) for L*, a*, and b* values for LL steaks (Figure 3). Although there was no diet effect on L* values on d 3, 4, and 8 of simulated retail display, values were greater on d 0, 1, 2, 5, 6, and 7 for HGP than CON steers. Although lower for HGP than CON steers on d 0 and 1 of simulated retail display, a* values were greater for HGP than CON steers on d 4, 5, 6, 7, and 8. Similarly, although they tended to be lower for HGP than CON steers on d 1 and 2 of simulated retail display, b* values tended to be greater for HGP than CON steers on d 4, 6, 7, and 8.

**Figure 4:**
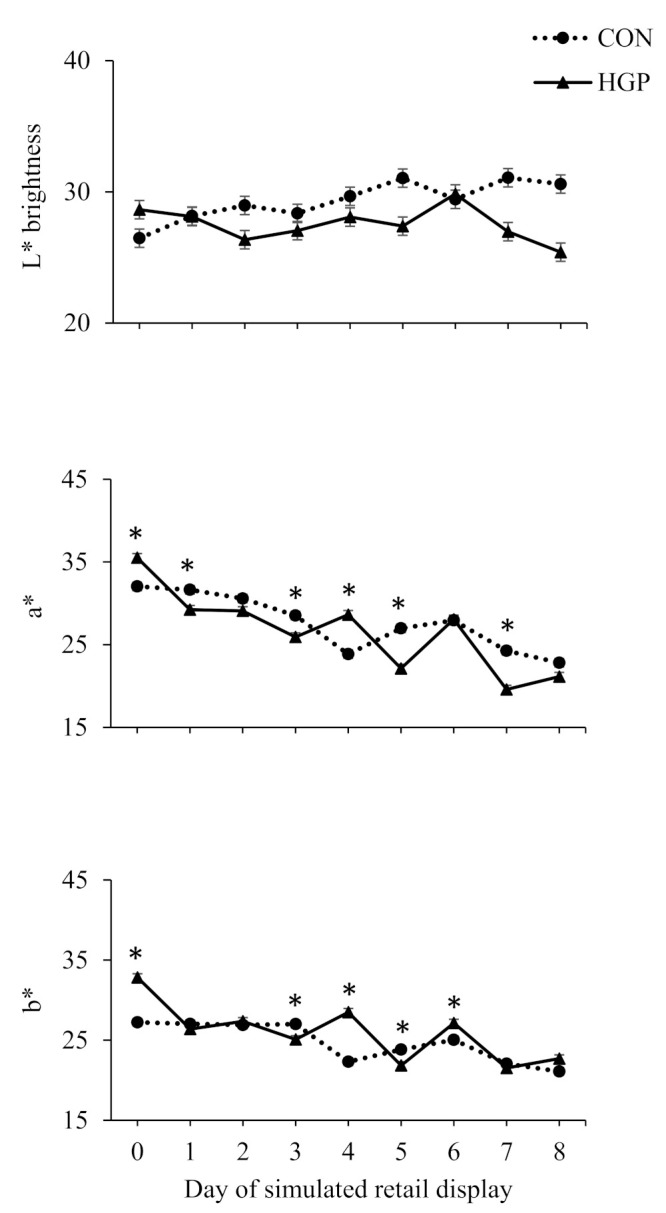
Hunter L*, a*, and b* color values over 8 d of simulated retail display for top round (*semimembranosus*) steaks from cattle fed a typical co-product-based finishing diet (CON) compared to a diet containing 58% (DM basis) grape pomace (HGP). L* color values pooled SEM = 0.6965. Diet, *p* = 0.18; day of simulated retail display, *p* < 0.01; diet × day of simulated retail display, *p* < 0.01. a* color values pooled SEM = 0.5049. Diet, *p* = 0.058; day of simulated retail display, *p* < 0.01; diet × day of simulated retail display, *p* < 0.01. b* color values pooled SEM = 0.4642. Diet, *p* = 0.03; day of simulated retail display, *p* < 0.01; diet × day of simulated retail display, *p* < 0.01. Error bars represent the standard deviation. * Significant difference between CON and HGP at *p* < 0.05.

There was also a diet × day of simulated retail display interaction (*p* < 0.01) for L*, a*, and b* values for SM steaks (Figure 4). Although greater for HGP than CON steers on d 0 of simulated retail display, L* values were greater for CON compared to HGP steers on d 2, 5, 6, and 7. As for a* values, they were greater for HGP than CON steers on d 0 and 4 of simulated retail display, whereas values were greater for CON compared to HGP steers on d 1, 3, 5, and 7. Similarly, although greater for HGP than CON steers on d 0, 4, and 6 of simulated retail display, b* values were greater for CON than HGP steers on d 3 and 5.

**Figure 5:**
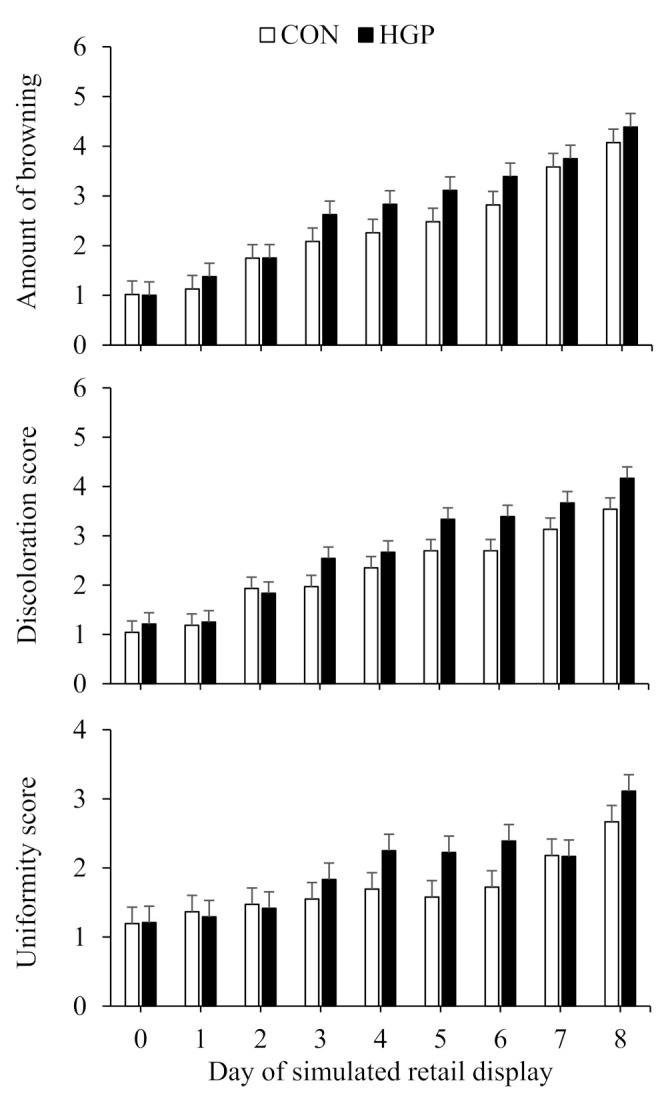
Browning, discoloration, and uniformity over 8 d of simulated retail display for striploin (*longissimus lumborum*) steaks from cattle fed a typical co-product-based finishing diet (CON) compared to a diet containing 58% (DM basis) grape pomace (HGP). Amount of browning pooled SEM = 0.2808. Diet, *p* = 0.01; day of simulated retail display, *p* < 0.01; diet × day of simulated retail display, *p* = 0.91. Discoloration score pooled SEM = 0.2385. Diet, *p* < 0.01; day of simulated retail display, *p* < 0.01; diet × day of simulated retail display, *p* = 0.66. Uniformity pooled SEM = 0.2460. Diet, *p* = 0.02; day of simulated retail display, *p* < 0.01; diet × day of simulated retail display, *p* = 0.60. Error bars represent the standard deviation.

**Figure 6:**
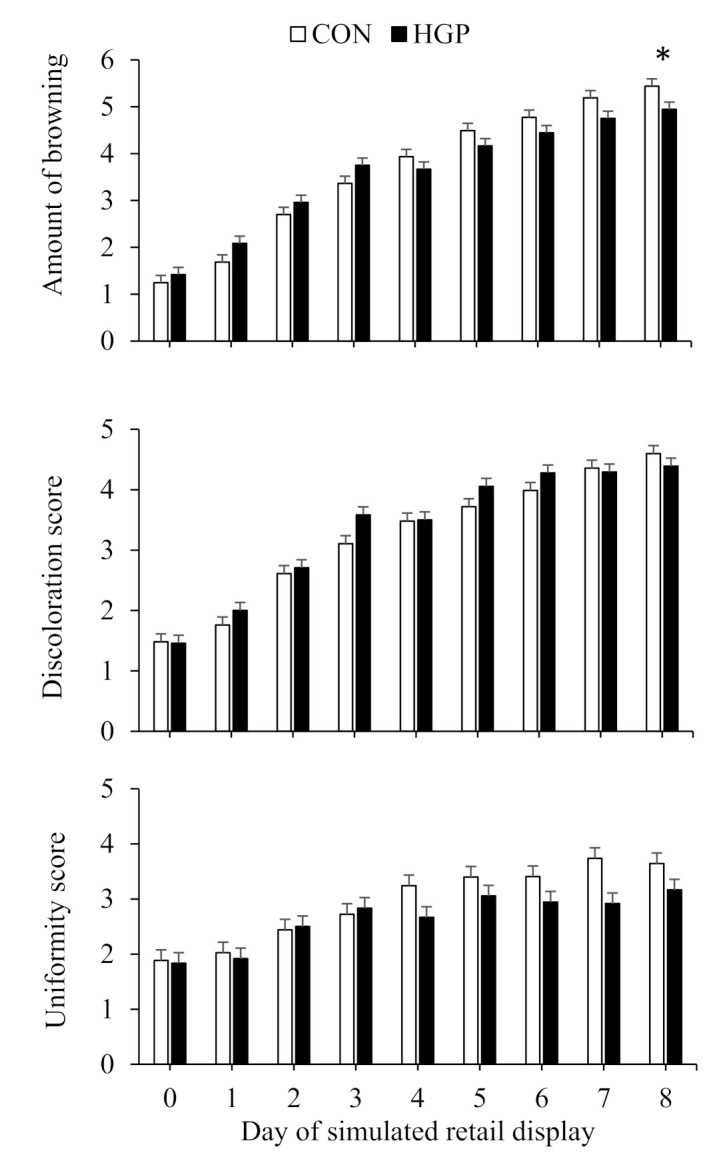
Browning, discoloration, and uniformity over 8 d of simulated retail display for top round (*semimembranosus*) steaks from cattle fed a typical co-product-based finishing diet (CON) compared to a diet containing 58% (DM basis) grape pomace (HGP). Amount of browning pooled SEM = 0.35. Diet, *p* = 0.01; day of simulated retail display, *p* < 0.01; diet × day of simulated retail display, *p* = 0.01. Discoloration score pooled SEM = 0.1373. Diet, *p* = 0.046; day of simulated retail display, *p* < 0.01; diet × day of simulated retail display, *p* = 0.25. Uniformity pooled SEM = 0.1988. Diet, *p* < 0.01; day of simulated retail display, *p* < 0.01; diet × day of simulated retail display, *p* = 0.26. Error bars represent the standard deviation. * Significant difference between CON and HGP at *p* < 0.05.

Overall, browning, and discoloration and uniformity scores of LL steaks increased (*p* < 0.01) over time (Figure 5). However, there was less (*p* ≤ 0.02) browning, discoloration, and uniformity for LL steaks from CON than HGP steers. Overall, discoloration and uniformity scores of SM steaks increased (*p* < 0.01) over time. There was a diet × day of simulated retail display interaction (*p* < 0.01) for browning for SM steaks; browning, which increased across diets with time was greater for CON than HGP steers on d 8 of simulated retail display (Figure 6). The discoloration score for SM steaks was greater (*p* = 0.046) for HGP than CON steers whereas the uniformity score was greater for CON than HGP steers. 

### 3.5. Fatty Acid Profile

The 15:0, 16:1 *c*9, 17:0, 17:1, total 18:1 *t*, 18:1 *t*12, 18:2 *t*10*c*12, and 20:1 concentration of LL steaks were greater (*p* ≤ 0.04) whereas the 14:1*c*9 concentration tended (*p* = 0.06) to be greater for CON than HGP steers (Table 5). However, the 18:2 *n-*6, 18:2 *c*9*t*11, 20:2 *c*11*c*14, 21:0, 20:3 *n-*6 concentration of LL steaks were greater (*p* ≤ 0.049) whereas the 18:0, 18:2 *t*9*t*12, and 24:1 concentration tended (0.052 ≤ *p* ≤ 0.07) to be greater for HGP than CON steers. Although total SFA, MUFA, and UFA concentrations, and the SFA:UFA ratio did not differ (*p* ≥ 0.17) across diets, total PUFA and CLA concentrations were greater (*p* < 0.01) in LL steaks for HGP than CON steers. The elongase and Δ^9^-desaturase enzyme activity indices for 16:0 and 18:0 were greater (*p* ≤ 0.03) for LL steaks from CON than HGP steers. 

The 10:0, 17:0, 17:1, 18:1 *t*12, total 18:1*t*, and 18:2 *t*10*c*12 concentration of SM steaks were greater (*p* ≤ 0.04), whereas the 15:0 (*p* = 0.06) and 20:1 (*p* = 0.08) concentration tended to be greater for CON than HGP steers (Table 6). However, the 18:2 *n-*6, 18:2 *c*9*t*11, 20:3 *n-*6, 20:4 *n-*6, and 22:5 *n-*3 concentration of SM steaks were greater (*p* ≤ 0.01), whereas the 20:0 concentration tended (*p* = 0.07) to be greater for HGP than CON steers. Although total SFA, MUFA, and UFA concentrations, and the SFA:UFA ratio did not differ (*p* ≥ 0.29) across diets, total PUFA and CLA concentrations were greater (*p* ≤ 0.02) in SM steaks from HGP than CON steers. The elongase, and Δ^9^-desaturase enzyme activity index for 18:0 did not differ (*p* ≥ 0.11) across diets; however, the Δ^9^-desaturase enzyme activity index for 16:0 tended (*p* = 0.07) to be greater for SM steaks from CON than HGP steers. 

## 4. Discussion

A key goal when formulating the experimental diets was for them to be iso-lipidic and iso-nitrogenous. This was achieved as the lipid content was 7.85 and 7.12% (DM basis), whereas the CP content was 15.5 and 16.0%, for the CON and HGP diets, respectively. However, energy supply was likely lower for the HGP than CON diet as reflected by the 63% decrease in dietary net energy of gain following the replacement of all starch sources including high-moisture corn and bakery waste with grape pomace. Grape pomace contains a high concentration of NDF and polyphenolics; therefore, this accounts for the NDF and polyphenolics content of the HGP diet being over 1-fold greater than the CON diet. There is an inverse relationship between dietary NDF content and dry matter intake [23]. Polyphenolics can also suppress nutrient digestibility [24]. Therefore, the greater NDF and polyphenolic content of the HGP compared to CON diet possibly compromised nutrient supply and, thus, growth performance in the present study. This is supported by the lower HCW, backfat thickness, and yield grade for HGP than CON steers. [25] also reported a decrease in metabolizable energy intake and dry matter digestibility that compromised BW gain and feed efficiency in steers fed a diet containing 30% (DM basis) ensiled grape marc. On the other hand, feeding up to 10% GP (DM basis) did not compromise growth performance and carcass traits in lambs [26,27]. The lower dietary inclusion level of GP (10 vs. 30 to 58% of diet DM) possibly accounts for the discrepancy between studies. This is supported by [28] who recently recommended a maximum inclusion level of 12% of diet DM after observing quadratic responses in DMI, ADG, and HCW when feeding increasing amounts of grape pomace in lamb diets. 

The oxidation of lipids by reactive oxygen species (ROS) is considered the major non-microbial cause of meat deterioration [29]. Therefore, the use of antioxidants to enhance oxidative stability of beef products, particularly through feeding strategies as it leads to cellular incorporation, is of great importance today. Meat content of unsaturated FA (UFA) is one of the major determinants of oxidative stability of beef products [30]. Because the PUFA content of LL and SM steaks was 49 to 75% greater, we also anticipated greater oxidative susceptibility for HGP compared to CON steers. However, feeding the HGP diet resulted in a desirable decrease in the MDA content of both LL and SM steaks on d 4, 6, and 8 of simulated retail display. Besides FA composition, other intrinsic factors, including the meat antioxidant content, influence lipid oxidation [29]. For instance, [27] reported a polyphenolic compound-induced decrease in the LL muscle MDA and ROS content, and increase in the activity of several antioxidative enzymes, including glutathione peroxidase, superoxide dismutase and total antioxidant capacity in lambs fed grape pomace. Similarly, [7] observed a decrease in the *longissimus* MDA and ROS content in lambs fed grape seed extract. Although it is unlikely that there is direct absorption of the polyphenolic compounds along the gut given their high molecular weight, the end-products of their degradation, which have a low molecular weight, could end up absorbed and transferred into tissue [31]. This is supported by the detection of different phenolic compounds including epicathechin and unknown phenolics in plasma of sheep dosed with a polyphenolic extract derived from several sources including grape peel and seed [32]. [33] also reported a 31% increase in muscle phenolic concentration following the addition of quebracho tannins to finishing lamb diets. Although not measured in the present study, feeding the HGP compared to the CON diet possibly increased the muscle phenolic content, thereby leading to the improvement in the antioxidative status. Therefore, this supports the feeding of grape pomace as a viable strategy to improve the shelf life of beef products, while also addressing consumer concerns about the use of synthetic antioxidants.

In addition to rancidity, a change in meat color due to oxidation is a major cause of consumer rejection of beef products at the point of purchase [34,35]. In the present study, overall lightness of LL steaks during simulated retail display was greater for HGP than CON steers. Redness, which is strongly correlated to myoglobin state, was also sustained over time suggesting a slower rate of myoglobin oxidation for HGP than CON steers possibly because of the antioxidant contribution of grape pomace. [33] also reported greater color stability over a 7-d aerobic storage period for *longissimus* muscle from quebracho tannin-fed compared to control lambs. On the other hand, feeding up to 20% grape pomace (DM basis) in lamb diets did not affect color attributes (L*, a* and b*) of *longissimus* muscle 24 h post-harvest [27,28]. [36] also did not observe changes in the lightness, redness and yellowness of *longissimus* muscle (3 d post-harvest) from Friesian calves feed supplemental grape pomace (10% of diet DM). The lower dietary grape pomace inclusion level (up to 20%) could account for the lack of an impact compared to the present study. It is also important to note that measurement of antioxidant status and color oxidation was 1 to 3 d postmortem, which could have been before benefits of any antioxidant effect are manifested. There was no change in overall lightness or an improvement in color stability for SM steaks from steers fed a high amount of grape pomace in the present study. Although reasons for this are not clear, the water holding capacity (WHC) of SM steaks was greater for CON that HGP steers; therefore, we had anticipated a darker color that is associated with a high WHC [35]. Browning, discoloration, and uniformity of both LL and SM steaks increased over time as expected. However, there was greater browning and discoloration of LL and SM steaks from HGP than CON steers, which was not consistent with objective color attributes. Besides differences in approach (visual vs. instrument appraisal) possibly accounting for the discrepancies, color evaluation is complex, with numerous factors including animal-to-animal variation, and inter- and intra-muscular effects influencing color perception [35]. 

Besides lipids, ROS also oxidize myofibril proteins, leading to a reduction in the WHC of beef products [29]. Therefore, the meat transfer of compounds from grape pomace including gallic acid and catechin could limit fluid loss by limiting structural changes in myofibril proteins including cross-linking and fragmentation, which compromise functionality. Although it did not result in changes in cook loss, fluid loss for LL steaks from HGP steers was 27.4% lower than from CON steers in the present study. [26] also reported an improvement in the WHC for *longissimus* muscle from lambs fed GP (up to 10% of diet DM). Therefore, although not evaluated in the present study, feeding GP could have also enhanced juiciness of LL steaks. As for SM steaks, there was an undesirable increase in both fluid and cook loss (40.7 and 32.4%, respectively) for HGP that CON steers, and the reasons for this are not clear. However, there is an inverse relationship between the rate and extent of meat pH decline and WHC, with muscle type being one of the factors that influences post-harvest pH changes [37,38]. Because it typically cools at a slower rate than *longissimus* muscle, SM muscle is more prone to low pH [37]. Meat pH was measured in the present study; however, it was not reported as some of the recorded data was misplaced. Therefore, it is not possible to deduce whether changes in muscle WHC were linked to pH. However, other factors, including sarcomere length, meat cooling temperature and rate, and heating rate and end point center temperatures during cooking, also influence fluid and cook losses [39,40]. 

Tenderness is one of the key palatability attributes that greatly influences the overall consumer beef eating experience [41]. Despite the additional 27 d needed to finish the HGP compared to CON steers, there was no diet effect on WBSF for both LL (3.31 vs. 3.62 kg for CON vs. HGP steers) and SM steaks (4.39 vs. 4.74 kg). In addition, the numerical differences between treatments for both muscle types were < 0.5 kg, the threshold for perceptible differences in tenderness by the average consumer when consuming meat at home [42]. [26] also did not observe changes in WBSF for *longissimus* muscle following the addition of GP (5 and 10% of diet DM) in ewe diets. On the other hand, [27] observed a decrease in WBSF (LL muscle) when ram diets contained GP (5 and 10% of diet DM), and this was attributed to a proanthocyanidin-induced decrease in collagen deposition, as protein expression of TGF-β, a promoter of collagen synthesis, was suppressed. However, muscle collagen content and profibrogenic cytokine expression were not quantified in the present study, and the study by [26]. *Longissimus* steaks from the present study would qualify to use the USDA AMS labeling claim “very tender” (<3.9 kg; [43]). However, the WBSF values for SM steaks were >4.4 kg, the threshold for use of the USDA AMS labeling claim “tender” across treatments. This was expected as tenderness is typically greater for LL than SM muscle [44]. 

Because of the reported associations between dietary fat consumption and health outcomes including CVD and cancer, consumers are increasingly taking into consideration the fat and FA composition of meat products when making purchasing decisions [45,46]. Therefore, current efforts to enhance the health value of beef lipids have focused on feeding strategies that reduce the meat content of SFA (e.g., 16:0) and specific *trans*-FA (TFA; e.g., 18:1 *t*-10), and increase the content of specific CLA (e.g., 18:2 *c*9*t*11) and TFA (e.g., 18:1 *t*-11) isomers and n-3 and n-6 PUFA (e.g., 18:3n-3 and 18:2 *n*-6) [45,46]. Although the capacity is limited, the dietary content of lipids, FA, and polyphenolic compounds are key factors that can be manipulated to enhance the FA profile of beef [24]. In the present study, diets were formulated to be isolipidic. However, the 18:2 *n*-6 content for the HGP diet was 81% greater than for the CON diet. This resulted in from the contribution of GP, which is rich in 18:2 *n*-6 [14]. A proportion of dietary PUFAs escapes ruminal biohydrogenation and ends up deposited in various organs and tissue. Polyphenolic compounds also limit biohydrogenation and increase ruminal escape of PUFA as they reduce the growth and activity of microbes including members of the genus *Butyrivibrio* [24]. In the current study, we observed a 58 to 80% increase in the 18:2 *n*-6 content of LL and SM muscle for HGP compared to CON steers. Although ruminal concentration was not measured, this suggests limited biohydrogenation of 18:2 *n*-6 possibly due to the greater polyphenolic content of the HGP than CON diet. Others [14,36] also reported a 25 to 49% increase in the 18:2 *n*-6 content of milk and LL muscle following the addition of 10 to 27% grape pomace (DM basis) in Friesian-Holstein cow and Friesian calf diets, respectively. This enrichment of 18:2 *n*-6 in muscle from finishing cattle fed grape pomace could be beneficial from a human health standpoint, as dietary replacement of SFA or total carbohydrate with 18:2 *n*-6 lowers the risk of CVD [46].

Biohydrogenation of PUFA, mainly 18:2 *n*-6 and 18:3 *n*-3, results in production of monounsaturated *cis*- and *trans*-FA (18:1 *c* and 18:1 *t*) and CLA isomers. The muscle content of 18:1 *c*9, which is the most common MUFA in the human diet, did not differ across dietary treatments in the present study. Similarly, dietary inclusion of either grape seed (11% of diet; as-fed) or grape pomace (up to 10% of diet DM) in ewe or calf diets did not result in changes in the 18:1 *c*9 concentration of ruminal fluid [47], milk [13] or *longissimus* muscle [36]. The total 18:1 *t* content of LL and SM muscle was greater for steers fed the CON than HGP diet in the present study. This was unexpected, as polyphenolic compounds have been reported to result in an increase in the muscle content of total 18:1 *t* at the expense of 18:0 [24]. The reasons for this are not clear; however, the impact of polyphenolic compounds on 18:1 *t* FA is isomer specific and is influenced by other factors including dietary forage:concentrate ratio. For instance, feeding supplemental quebracho powder (4% dietary tannins; DM basis) to sheep offered either a high forage or high concentrate diet had no effect on the 18:1 *t*-11 content of *longissimus* muscle [48]. However, the *longissimus* 18:1 *t*-10 content was greater for sheep fed the high concentrate diet with the tannin supplement compared to without, whereas, there was no supplement effect on the high forage diet [48]. 18:1-*t* FA intake also influences health outcomes in an isomer specific manner; for instance, increased 18:1 *t*-11 but not 18:1 *t*-10 intake has been associated with enhance cardiovascular health [49]. Because the peaks for all isomers were not resolved in the present study, it is not possible to fully deduce the potential implications of feeding the HGP diet on muscle accumulation and, ultimately, health outcomes. 

The 18:0 content of LL muscle tended to be greater for HGP than CON steers, whereas there was no diet effect for SM muscle in the current study. [47] also observed an increase in the 18:0 concentration of ruminal fluid following the addition of grape seed to ewe diets. However, [36] did not observe changes in the 18:0 content of LL muscle from calves fed grape pomace. This lack of the anticipated decrease in ruminal fluid and muscle 18:0 content could be due to the 18:2 *n*-6 contribution of grape seed that increases dietary PUFA content and, thus substrate for biohydrogenation. In the present study, there was also no diet effect on total SFA and MUFA; however, feeding the HGP than CON diet led to an increase in total PUFA in both LL and SM muscle. Besides 18:2 *n*-6, this increase in total PUFA was in part due to the greater contribution of 18:2 *c*9*t*11, which makes up >80% of total CLA in ruminant muscle [50]. Others [14,47] also reported an increase in the 18:2 *c*9*t*11 content of ruminal fluid and milk after adding grape pomace to diets. This is suggestive of increased intestinal absorption of 18:2 *c*9*t*11 and possibly *de novo* synthesis from 18:1 *t*11 in tissue, a process regulated by Δ^9^—desaturase [51]. Estimated Δ^9^—desaturase 18 activity, which did not differ across dietary treatments for SM muscle, was greater for CON than HGP steer in LL muscle, and this was not consistent with the noted change in 18:2 *c*9*t*11 content as dietary polyphenolic content increased. This contrasts with [52] who observed increased muscle Δ^9^—desaturase protein expression and total 18:1 *t* and 18:2 *n*-6 content in lambs fed tannins. However, it is not possible to determine the absolute contribution of *de novo* synthesis and intestinal absorption to muscle 18:2 *c*9*t*11 content. In addition, estimation of Δ^9^—desaturase 18 activity using the ratio of product to substrate might not be as accurate as direct measurement of muscle protein expression, thereby possibly accounting for the discrepancies between studies. On the other hand, the milk and meat content of 18:2 *c*9*t*11, 18:1 *t*-10 and 18:1 *t*-11 were reported [13,36] not to change when diets contained up to 10% grape pomace. This possibly was because of the low dietary inclusion level. The muscle content of 18:2 *t*10*c*12, which makes up 5% of total CLA in ruminant muscle [50], was lower for HGP than CON steers in the present study. Therefore, the divergent responses in muscle 18:2 *c*9*t*11 and 18:2 *t*10*c*12 content suggests polyphenolic-induced changes in ruminal biohydrogenation pathways that could have led to increased accumulation of 18:1 *t*-11 at the expense of 18:1 *t*-10 in muscle for HGP steers [53]. Total muscle CLA was also greater for HGP than CON, and this could be beneficial from a human nutrition standpoint as increased intake of CLA has been suggested to improve energy and bone metabolism, immune and inflammation status, and inhibit mutagenesis, among other factors [45]. Besides CLA, feeding the HGP compared to CON diet also resulted increased accumulation of long chain PUFA including 20:3 *n*-6, 20:4 *n*-6, and 22:5 *n*-3 in LL and SM muscle. Estimated elongase activity was also greater in LL muscle for steers fed the HGP than CON diet. [13] also reported an increase in the milk content of 20:3 *n*-6 and 20:4 *n*-6 following the addition of grape pomace to dairy ewe diets. This increase is desirable as long chain PUFA like 22:5 *n*-3 have anti-atherosclerotic and cardio-protective properties, and enhance brain, neural and visual development and function [45]. Therefore, given the noted changes in the muscle content of several bioactive FA including 18:2 *c*9*t*11, total CLA and PUFA, feeding grape pomace to finishing cattle could be an effective strategy to enhance the health value of beef lipids. However, it is worthwhile noting that potential benefits ultimately are dependent on FA intake, which is not only a function of muscle fatty acid composition as reported, but also muscle fat content and total muscle/meat consumption.

## 5. Conclusions

Feeding a high amount of grape pomace to finishing cattle resulted in a decrease in hot carcass weight, backfat thickness, and yield grade due to an energy deficiency caused by the decrease in dietary starch content and increase in fiber and polyphenolic compounds content. However, the high dietary polyphenolic compounds content was beneficial as it resulted in a desirable decrease in lipid oxidation of LL and SM muscle during 8-d simulated retail display, despite the increase in PUFA content. Dietary inclusion of a high amount of grape pomace to finishing diets also led to increased muscle accumulation of several FA linked to positive health outcomes in humans including 18:2 *n*-6, 18:2 *c*9*t*11, total CLA, and total PUFA, and this possibly was also due to polyphenolic compound-induced changes in biohydrogenation pathways in the rumen. This research demonstrated that although feeding a high amount of grape pomace to finishing cattle could limit growth performance, it could be an effective strategy to enhance the sensory quality and fatty acid profile of meat, which are key in increasing consumer acceptability of beef. 

## Figures and Tables

**Table 1:** Diet ingredient and chemical composition.

	Diet	
Item	Control (CON)	Grape Pomace (HGP)
Ingredient, % of dry matter		
Straw	1.80	4.61
Cattle finisher supplement ^1^	1.71	2.88
Wheat mill run	9.02	34.7
Grape pomace	7.54	57.8
Apple pomace	7.58	_
High moisture corn	22.5	_
Wet corn gluten	21.0	_
Barley grain, dry rolled	11.2	_
Bakery waste	9.02	_
Whey	5.69	_
Optaflexx	2.93	_
Chemical analysis		
Dry matter (DM), %	50.6 ± 1.79	54.7 ± 3.44
Organic matter (OM), % of DM	93.2 ± 1.30	87.0 ± 0.80
Neutral detergent fiber (NDF), % of DM	20.7 ± 2.52	47.9 ± 3.62
Acid detergent fiber (ADF), % of DM	14.1 ± 3.11	35.8 ± 3.21
Crude fat (CF), % of DM	7.85 ± 0.779	7.12 ± 0.600
Crude protein (CP), % of DM	15.5 ± 1.85	16.0 ± 1.15
Net energy of gain, Mcal/kg	1.34 ± 0.07	0.49 ± 0.13
Phenolic compounds, % of DM		
Total phenols	1.60 ± 0.180	3.19 ± 0.404
Free phenols	0.96 ± 0.067	0.43 ± 0.082
Tannins	0.94 ± 0.271	2.77 ± 0.379
Nontannins	0.66 ± 0.091	0.42 ± 0.057

^1^ Supplement contained CP, 12.0%; CF, 5.00%; Crude fiber, 15.0%; Ca, 1.65%; K, 0.60%; P, 0.28%; NaCl, 0.50%; and Vitamin A, 907 IU/kg.

**Table 2:** Diet fatty acid composition (g/100 g of fatty acid methyl esters).

	Diet	
Fatty Acid	Control (CON)	Grape Pomace (HGP)
Myristic (14:0)	0.962 ± 0.6022	0.273 ± 0.0869
Pentadecanoic (15:0)	0.174 ± 0.0977	0.096 ± 0.0308
Palmitic (16:0)	15.7 ± 4.25	12.8 ± 1.03
Palmitoleic (16:1)	0.862 ± 0.5526	0.291 ± 0.0201
Margaric (17:0)	0.368 ± 0.2366	0.150 ± 0.0365
Stearic (18:0)	6.53 ± 3.671	3.22 ± 0.567
Oleic (18:1)	26.5 ± 5.91	17.6 ± 1.14
Linoleic (18:2*n*-6)	30.2 ± 6.99	54.6 ± 2.62
α-Linolenic (18:3*n*-3)	3.06 ± 1.400	3.32 ± 1.282

**Table 3:** Carcass characteristics for cattle fed a typical co-product-based finishing diet (CON) compared to a diet containing 58% (DM basis) grape pomace (HGP).

	Diet			
Variable	CON	HGP	SEM	*p*-Value
HCW, kg	343	301	5.55	<0.01
Dressing %	55.6	54.2	0.93	0.30
Backfat thickness, cm	0.836	0.323	0.0401	<0.01
Preliminary yield grade	2.85	2.26	0.044	<0.01
Ribeye area, cm^2^	69.0	65.8	1.66	0.14
Kidney, Pelvic, and Heart, %	4.63	4.25	0.177	0.15
Finally, yield grade	3.32	2.94	0.094	0.04
Marbling	529	500	18.1	0.27

**Table 4:** Fluid and cook loss, and Warner-Bratzler shear force for striploin (*longissimus lumborum*) and top round (*semimembranosus*) steaks from cattle fed a typical co-product-based finishing diet (CON) compared to a diet containing 58% (DM basis) grape pomace (HGP).

	Diet			
Variable	CON	HGP	SEM	*p*-Value
Striploin (*longissimus lumborum*)				
Fluid loss, %	1.829	1.327	0.1767	0.057
Cook loss, %	25.3	25.3	1.32	0.98
Warner-Bratzler shear force, kg	3.31	3.62	0.226	0.35
Top round (*semimembranosus*)				
Fluid loss, %	1.235	1.738	0.1033	<0.01
Cook loss, %	26.8	35.5	1.82	<0.01
Warner-Bratzler shear force, kg/cm^3^	4.39	4.74	0.239	0.30

**Table 5:** Fatty acid composition (g/100 g of fatty acid methyl esters) of striploin (*longissimus lumborum*) steaks from cattle fed a typical co-product-based finishing diet (CON) compared to a diet containing 58% (DM basis) grape pomace (HGP).

	Diet			
Variable	CON	HGP	SEM	*p*-Value
10:0	0.041	0.039	0.0045	0.77
12:0	0.063	0.059	0.0063	0.59
13:0	0.029	0.029	0.0078	0.99
14:0	2.79	2.23	0.239	0.11
14:1 *c*9	0.726	0.546	0.0643	0.06
15:0	0.397	0.283	0.0361	0.04
15:1	0.0570	0.0463	0.0110	0.27
16:0	22.0	20.1	1.66	0.44
16:1 *c*9	3.14	2.36	0.238	0.03
17:0	1.04	0.68	0.072	<0.01
17:1	0.661	0.396	0.0719	0.02
18:0	12.2	15.6	1.17	0.052
18:1 *t*	4.35	1.36	0.437	<0.01
18:1 *c*9	33.4	32.5	2.35	0.80
18:1 *t*12	1.82	1.21	0.117	<0.01
18:2 *n*-6	3.86	6.10	0.461	<0.01
18:2 *n*-4	0.116	0.115	0.0111	0.95
18:3*n*-3	0.366	0.365	0.0262	0.96
18:2 *c*9*t*11	0.213	0.419	0.0312	<0.01
18:2 *t*9*t*12	0.273	0.340	0.0249	0.07
18:2 *t*10*c*12	0.0531	0.0144	0.00577	0.04
20:2 *c*11*c*14	0.0542	0.0747	0.00699	0.049
20:0	0.0060	0.0197	0.00610	0.93
20:1	0.0540	0.0056	0.00534	0.03
21:0	0.041	0.088	0.00820	0.03
23:0	0.0040	0.0147	0.00566	0.11
24:1	0.0240	0.0865	0.01230	0.06
20:3 *n*-6	0.160	0.227	0.0215	0.04
20:4 *n*-6	0.540	0.742	0.0986	0.16
22:5 *n*-3	0.0479	0.0669	0.01114	0.24
SFA ^1^	38.6	39.2	3.06	0.89
MUFA ^2^	44.2	38.5	2.94	0.19
PUFA ^3^	5.68	8.46	0.623	<0.01
UFA ^4^	49.9	47.0	3.37	0.55
SFA:UFA	0.77	0.83	0.029	0.17
Total CLA ^5^	0.539	0.772	0.0581	<0.01
Δ^9^—desaturase 16 ^6^	12.5	10.5	0.48	0.01
Δ^9^—desaturase 18 ^7^	73.2	67.6	1.23	0.03
Elongase ^8^	64.6	68.4	0.54	<0.01

^1^ SFA = Σ10:0, 11:0, 12:0, 13:0, 14:0, 15:0, 16:0, 17:0, 18:0, 20:0, 21:0, and 23:0; ^2^ MUFA = Σ14:1*c*9, 15:1, 16:1*c*9, 17:1, 18:1*t*, 18:1*c*9, 18:1*t*12, 20:1, and 24:1; ^3^ PUFA = Σ18:2*n*-6, 18:2*n*-4, 18:3*n*-3, 18:2 *c*9*t*11, 18:2 *t*9*t*12, 18:2 *t*10*c*12, 20:2 *c*11*c*14, 20:3*n*-6, 20:4*n*-6, and 22:5*n*-3; ^4^ UFA = MUFA + PUFA; ^5^ CLA = Σ18:2 *c*9*t*11, 18:2 *t*9*t*12, and 18:2 *t*10*c*12; ^6^ Δ^9^—desaturase 16 = 100[(16:1*c*9)/(16:1*c*9 + 16:0)]; ^7^ Δ^9^—desaturase 18 = 100[(18:1*c*9)/(18:1*c*9 + 18:0)]; ^8^ Elongase = 100[(18:0 + 18:1*c*9)/(16:0 + 16:1*c*9 + 18:0 + 18:1*c*9)].

**Table 6:** Fatty acid composition (g/100 g of fatty acid methyl esters) of top round (*semimembranosus*) steaks from cattle fed a typical co-product-based finishing diet (CON) compared to a diet containing 58% (DM basis) grape pomace (HGP).

	Diet			
Variable	CON	HGP	SEM	*p*-Value
10:0	0.124	0.064	0.0141	0.01
12:0	0.0334	0.0319	0.00478	0.82
13:0	_	_	_	_
14:0	2.07	1.82	0.213	0.47
14:1 *c*9	0.517	0.479	0.0621	0.67
15:0	0.297	0.218	0.0284	0.06
15:1	0.062	0.139	0.0390	0.27
16:0	20.3	19.2	1.86	0.68
16:1 *c*9	2.69	2.30	0.263	0.35
17:0	0.976	0.627	0.0793	<0.01
17:1	0.677	0.379	0.0531	<0.01
18:0	11.9	12.8	1.39	0.66
18:1 *t*	3.99	1.78	0.354	<0.01
18:1 *c*9	32.9	30.9	2.73	0.62
18:1 *t*12	1.99	1.45	0.161	0.03
18:2 *n*-6	4.29	7.74	0.737	<0.01
18:2 *n*-4	0.083	0.106	0.0097	0.11
18:3 *n*-3	0.332	0.288	0.0307	0.32
18:2 *c*9*t*11	0.178	0.350	0.0294	<0.01
18:2 *t*9*t*12	0.268	0.317	0.0282	0.23
18:2 *t*10*c*12	0.0227	0.0000	0.00515	<0.01
20:2 *c*11*c*14	0.0208	0.0633	0.00767	0.28
20:0	0.0000	0.0118	0.00446	0.07
20:1	0.0121	0.000	0.00469	0.08
21:0	0.0211	0.0900	0.01065	0.67
23:0	0.0054	0.0277	0.00994	0.76
24:1	0.0079	0.0645	0.01400	0.86
20:3 *n*-6	0.141	0.279	0.0343	<0.01
20:4 *n*-6	0.52	1.12	0.139	<0.01
22:5 *n*-3	0.0110	0.0655	0.01086	<0.01
SFA^1^	35.8	35.0	3.17	0.85
MUFA^2^	42.8	37.5	3.47	0.29
PUFA^3^	5.87	10.3	0.980	<0.01
UFA^4^	48.7	47.9	4.25	0.89
SFA:UFA	0.735	0.735	0.0243	0.99
Total CLA^5^	0.468	0.666	0.0576	0.02
Δ^9^—desaturase 16 ^6^	11.8	10.5	0.48	0.07
Δ^9^—desaturase 18 ^7^	73.6	70.7	1.87	0.29
Elongase ^8^	66.0	76.7	0.92	0.20

^1^ SFA = Σ10:0, 11:0, 12:0, 13:0, 14:0, 15:0, 16:0, 17:0, 18:0, 20:0, 21:0, and 23:0; ^2^ MUFA = Σ14:1*c*9, 15:1, 16:1*c*9, 17:1, 18:1*t*, 18:1*c*9, 18:1*t*12, 20:1, and 24:1; ^3^ PUFA = Σ18:2*n*-6, 18:2*n*-4, 18:3*n*-3, 18:2 *c*9*t*11, 18:2 *t*9*t*12, 18:2 *t*10*c*12, 20:2 *c*11*c*14, 20:3*n*-6, 20:4*n*-6, and 22:5*n*-3; ^4^ UFA = MUFA + PUFA; ^5^ CLA = Σ18:2 *c*9*t*11, 18:2 *t*9*t*12, and 18:2 *t*10*c*12; ^6^ Δ^9^—desaturase 16 = 100[(16:1*c*9)/(16:1*c*9 + 16:0)]; ^7^ Δ^9^—desaturase 18 = 100[(18:1*c*9)/(18:1*c*9 + 18:0)]; ^8^ Elongase = 100[(18:0 + 18:1*c*9)/(16:0 + 16:1*c*9 + 18:0 + 18:1*c*9)].

## Data Availability

Presented data is available upon reasonable request from the corresponding author.
